# Lung cancer screening: assessment of health literacy and readability of online educational resources

**DOI:** 10.1186/s12889-018-6278-8

**Published:** 2018-12-07

**Authors:** Kevin Haas, Christie Brillante, Lisa Sharp, Ahmed K. Elzokaky, Mary Pasquinelli, Lawrence Feldman, Kevin L. Kovitz, Min Joo

**Affiliations:** 1 University of Illinois at Chicago, 840 S. Wood St., CSB 915, MC 719, Chicago, IL 60612 USA; 2 University of Illinois College of Pharmacy, 463 Westside Research Office Bldg. 1747 West Roosevelt Road, Chicago, IL 60608 USA; 3 University of Illinois at Chicago, 840 S Wood Street, 820-E CSB, MC 713, Chicago, IL 60612 USA

**Keywords:** Health literacy, Lung cancer screening, Patient education

## Abstract

**Background:**

Lung cancer screening can reduce mortality but can be a complex, multi-step process. Poor health literacy is associated with unfavorable outcomes and decreased use of preventative services, so it is important to address barriers to care through efficient and practical education. The readability of lung cancer screening materials for patients is unknown and may not be at the recommended 6th grade reading level set by the American Medical Association. Our goals were to: (1) measure the health literacy of a lung cancer screening population from an urban academic medical center, and (2) examine the readability of online educational materials for lung cancer screening.

**Methods:**

We performed a retrospective cross sectional study at a single urban academic center. Health literacy was assessed using three validated screening questions. To assess the readability of educational materials, we performed a Google search using the phrase, “What is lung cancer screening?” and the Flesch-Kincaid Grade Level (FKGL) formula was used to estimate the grade level required to understand the text.

**Results:**

There were 404 patients who underwent lung cancer screening during the study period. The prevalence of inadequate/marginal health literacy was 26.7–38.0%. Fifty websites were reviewed and four were excluded from analysis because they were intended for medical providers. The mean FKGL for the 46 websites combined was 10.6 ± 2.2.

**Conclusions:**

Low health literacy was common and is likely a barrier to appropriate education for lung cancer screening. The current online educational materials regarding lung cancer screening are written above the recommended reading level set by the American Medical Association.

## Background

Lung cancer is the leading cause of cancer related deaths in the United States with a worse prognosis among the underserved and ethnic minority populations [1–3]. Early diagnosis is associated with improved mortality, yet more than 50% of lung cancer is diagnosed at an advanced stage [4, 5]. The National Lung Cancer Screening Trial (NLST) demonstrated a 20% mortality reduction from lung cancer using annual computed tomography to screen high risk patients [3]. Like much of cancer research however, the sample was 90% white [6–11]. Thus little is known about the impact of sociodemographic factors such as race, ethnicity, income, education, and health literacy on lung cancer screening (LCS) outcomes. This is important since a low socioeconomic status (SES) limits a patients’ ability to follow cancer screening recommendations and often results in finding more advanced disease [3, 12].

Primary care physicians (PCP) and pulmonologists play a vital role in LCS and comprise the majority of ordering physicians [13]. Counseling for lung cancer screening should involve a shared decision making discussion between the ordering physician and their patient [14]. The lung cancer screening process can be complex and involve several diagnostic pathways, referrals, and procedures the patient will need to consider if an abnormality is found. The discussion should include the risks and benefits of screening, appropriate follow up, implications of false positive findings, radiation exposure, and smoking cessation [14]. The ordering physician is ultimately responsible for patient education about lung cancer screening. Time constraints and limited resources are known barriers to LCS making patient education in a busy clinic environment a challenge [13, 15–18]. This can lead to many patients using the internet for their health information [19]. Improved educational modalities, especially for low literacy patients, are needed to improve the screening process.

The Lung Cancer Alliance estimates there are over 530 lung cancer screening centers in the U.S. with little attention directed at ensuring educational materials match the literacy level of participants. In general, prior studies have found health related educational materials often exceed the American Medical Association’s (AMA) recommended 6th grade reading level making the information incomprehensible to many patients [20–23]. Health literacy is defined as the degree to which individuals have the capacity to obtain, process, and understand health information needed to interpret quantitative information and make appropriate decisions [24, 25]. Only 12% of Americans have proficient health literacy with low health literacy being more prevalent in the elderly, populations with low SES, the uninsured, and publicly insured patients [24–26].

Understanding and addressing low health literacy in LCS patients is vital to improve outcomes and maintain patient autonomy. In the NLST, 27% of patients who received the initial screening CT scan were defined as having a positive scan but only 3.8% of these patients had lung cancer [3]. Providers need to make patients aware that false positive findings are common and up to 25% of invasive procedures may be performed on cancer-free patients [27]. Patient education is integral to understand this complex process in addition to the possible diagnostic dilemmas that may arise.

Little is known about the literacy level of current educational materials for LCS. The goals of our study were to: 1) assess the health literacy of patients participating in a lung cancer screening program within an urban academic institution, and 2) examine the readability of online educational materials for lung cancer screening.

## Methods

We performed a retrospective cross sectional study at a single urban academic medical center with an established lung cancer screening program. Patients who were eligible for LCS were required to have a shared decision making discussion with their ordering provider (e.g. primary care physicians, pulmonologists, and nurse practitioners) during a clinic visit. However, no standardized decision aids were used during the study period. Only those who agreed to lung cancer screening between June 2015 and June 2017 were included in the study. All patients met criteria for screening based on the United States Preventive Services Task Force recommendations. The most common referral sources were within the center and from affiliated health care centers in the community.

The study protocol was determined to be exempt by the Institutional Review Board (IRB).

### Assessment of health literacy

Participants independently completed a baseline clinic intake form, which included demographic information and a validated three item health literacy screen [28–31]. The response to each health literacy screening question was based on a 5-point Likert scale. The response options for questions one and three were as follows: (1/never), (2), (3), (4), (5/always). The response options for question two was as follows: (1/unsure), (2), (3), (4), (5/very sure). The items, shown below, were designed to overcome underreporting and social stigma by asking “how often” rather than “if” participants had a problem [28, 32]. In addition, the literacy screen was validated against the Short Test of Functional Health Literacy in Adults (S-TOFHLA) and the Rapid Estimate of Adult Literacy in Medicine (REALM) to identify patients with inadequate or marginal health literacy [28–31]. This brief screen was selected as opposed to the TOFHLA or REALM to decrease time burden.

Q1. How often do you have problems learning about your medical condition because of difficulty understanding written information? (sensitivity 57%, specificity 78%) [28].

Q2. How sure are you filling out medical forms by yourself? (sensitivity 77–80%, specificity 74–77%) [28, 30].

Q3. How often do you have someone help you read hospital materials? (sensitivity 54–73%, specificity 83%) [28, 31].

Health literacy was determined by analyzing responses independently as single item screening questions and in combination when completed. Importantly, questions 2 and 3 have been validated in separate studies as single item screening questions to detect low health literacy with comparable sensitivity and specificity [30, 31]. Question 1, though independently effective, had a lower sensitivity than questions 2 and 3 [28].

The patients were categorized into two groups; inadequate/marginal or adequate health literacy. Similar to prior studies, inadequate health literacy was defined as a reading level ≤ 6th grade and marginal health literacy corresponded to a reading level between 7th and 8th grade [30]. The reading level was assessed based on their response to the validated health literacy screening questions. A cut off of ≥3 for question 1 and question 3 and a cutoff of ≤3 for question 2 indicated inadequate/marginal health literacy.

### Statistical analysis

Those who responded to at least one health literacy question (responders) were compared to those who did not complete any literacy screening items (non-responders) to explore if there were demographic differences between the groups. Chi square and t-test analyses were used on categorical and interval data, respectively.

Descriptive statistics were calculated using SPSS Statistics for Mac version 24 (New York, USA). A *p*-value of ≤0.05 was used to determine statistical significance on all analyses.

### Assessment of online educational materials

As a separate analysis, we evaluated the readability level of online educational materials regarding lung cancer screening available to the general population. The Flesch-Kincaid Grade Level (FKGL) readability formula is an objective measure of language complexity [32]. The formula is based on the number of words per sentence and the number of syllables per word. It estimates the U.S. grade level required to understand written material. For example, a FKGL score of 6 indicates the material is written at a 6th grade reading level. The Flesch-Kincaid formula has been used in several studies of readability and demonstrates a high correlation with other readability scales [32]. The formula is as follows:

$$ 0.39\left(\mathrm{total}\kern0.5em \mathrm{words}/\mathrm{total}\kern0.5em \mathrm{sentences}\right)+11.8\left(\mathrm{total}\kern0.5em \mathrm{syllables}/\mathrm{total}\ \mathrm{words}\right)-15.59 $$

Using the phrase “What is lung cancer screening?” we performed a Google search on November 17, 2017 for patient focused educational materials from English language websites. Account information and location services were disabled for the search. Web page navigation, copyright notices, postal addresses, phone numbers, uniform resource locaters, disclaimers, date stamps, author information, citations, references, feedback questionnaires, and hyperlinks were not included in the analysis. The first 50 search results were reviewed which included content containing at least 300 words specifically addressing lung cancer screening. The websites contained information on eligibility, imaging tests, cost, risks, and benefits of testing. The FKGL was analyzed using the built in readability tools on Microsoft Word software [23, 33, 34]. The sites were categorized into type of institution providing the content (e.g. academic centers, lung cancer organizations, etc.) and the mean grade level was analyzed per category.

## Results

There were 404 patients who underwent lung cancer screening during the study period, with the majority being referred by primary care physicians (74%) followed by pulmonologists (19%). Demographic characteristics are presented in Table 1. The population was predominantly black (70.0%) with 51.5% being men.

**Table 1:** Patient demographics based on responders and non-responders to at least one health literacy question

	Total	Non-Responders	Responders	*P* Value
	N (%)	N (%)	N (%)	
Total	404	204	200	
Sex				
Males	208 (51.5)	108 (52.9)	100 (50.0)	*p* = 0.554
Race				
Black	283 (70.0)	143 (70.0)	140 (70.0)	*p* = 0.943
White	71 (17.6)	37 (18.1)	34 (17.0)	
Non-white Hispanic	44 (10.9)	21 (10.3)	23 (11.5)	
Asian/Hawaiian/Pacific Islander	5 (1.2)	3 (0.15)	2 (1.0)	
No Answer	1 (0.2)	0 (0)	1 (0.5)	
Marital Status				
Single	138 (34.1)	71 (34.8)	67 (33.5)	*p* = 0.001
Married	88 (21.8)	30 (14.7)	58 (29.0)	
Divorced/Separated/Widowed	95 (23.5)	27 (13.2)	68 (34.0)	
Unknown	83 (20.5)	76 (37.3)	7 (3.5)	
Age				
Mean ± SD		63.95 ± 6.22	63.02 ± 5.22	*p* = 0.104
Age range				
55–59	124 (30.7)	62 (30.4)	62 (31.0)	*p* = 0.010
60–64	127 (31.4)	63 (30.9)	64 (32.0)	
65–69	89 (22.0)	39 (19.1)	50 (25.0)	
70–74	45 (11.1)	23 (11.3)	22 (11.0)	
75–80	19 (4.7)	17 (8.3)	2 (1.0)	
Language				
English	373 (92.3)	190 (93.1)	183 (91.5)	*p* = 0.603
Other	22 (5.4)	13 (6.4)	9 (4.5)	
No Answer	8 (2.0)	1 (0.5)	7 (3.5)	
Income				
Wish not to answer (selected choice)	27 (6.7)	1 (0.5)	26 (13.0)	*
≤ $15,000/Yr	95 (23.5)	6 (2.9)	89 (44.5)	
$15,000–$30,000/Yr	29 (7.2)	3 (1.5)	26 (13.0)	
≥ $30,000/Yr	24 (5.9)	0 (0.0)	24 (12.0)	
No Answer	229 (56.7)	194 (95.1)	35 (17.5)	
Insurance				
Medicare	134 (33.2)	63 (30.8)	71 (35.5)	*p* = 0.716
Medicaid	184 (45.5)	93 (45.5)	91 (45.5)	
Private	37 (9.2)	21 (10.3)	16 (8.0)	
Self Pay/Other/Blank	49 (12.1)	27 (13.2)	22 (11.0)	

*comparison not performed due to high non-response rate

Half of the participants (*n* = 200, 49.5%) completed at least one health literacy question and were defined as ‘responders’. Only 36% (146/404) answered all three screening questions. Of responders, 44.5% (*n* = 89) reported an annual income of ≤$15,000 and 81% (*n* = 162) had public insurance. It should be noted non-responders were less likely than responders to reveal their marital status or income, and were more likely to be elderly.

The results of the health literacy questions are presented in Table 2. The prevalence of inadequate/marginal health literacy was 26.7–38.0%, depending on the item(s) used.

**Table 2:** Inadequate/marginal health literacy based on responses to specific screening questions

	Q1 only	Q2 only	Q3 only	Q2 or Q3	Q1 or Q2 or Q3
Number of responses	178	173	176	189	200
Number of responses indicating inadequate or marginal literacy (%)	30 (16.8)	47 (27.2)	47 (26.7)	65 (34.4)	76 (38.0)

Q1. How often do you have problems learning about your medical condition because of difficulty understanding written information?(Score ≥ 3 indicates inadequate/marginal health literacy)

Q2. How sure are you filling out medical forms by yourself?(Score ≤ 3 indicates inadequate/marginal health literacy)

Q3. How often do you have someone help you read hospital materials? (Score ≥ 3 indicates inadequate/marginal health literacy)

### Readability of online educational material

Fifty websites were reviewed and four were excluded from analysis because they were intended for medical providers. The readability level of the 46 websites are presented in Table 3. The mean FKGL for the 46 websites combined was 10.6 ± 2.2. Most websites were from academic medical centers (39%). Only one website, from an academic center, was written at a 6th grade reading level. In total, ten web sites (21.7%) were at or below an 8th grade reading level. Government web sites had the lowest mean FKGL (8.5 ± 0.96) and public websites had the highest (12.7 ± 2.63).

**Table 3:** Flesch-Kincaid analysis of lung cancer educational materials

Source of Web site	N	Grade level range	Average grade level and standard deviation
Academic Medical Centers	18	6.4–15.6	10.7±2.10
Non-academic Hospital System or Network^a^	8	7.1–13.1	10.1±2.15
Lung Cancer organizations	5	7.4–13.9	10.1±2.18
Government^b^	3	7.5–9.8	8.5±0.96
Community/Private Hospitals	9	9.0–13.6	11.3±1.63
Public Web sites^c^	3	10.1–16.3	12.7±2.63
Total	46	6.4–16.3	10.6±2.20

^a^A hospital system is two or more hospitals owned, leased, sponsored, or contract managed by a central organization, while a network is a group of hospitals, physicians, other providers, insurers and/or community agencies that coordinate and deliver a broad spectrum of services to their community [56]

^b^Centers for Disease Control and Prevention, United States Preventive Services Task Force, National Cancer Institute

^c^Wikipedia, Radiologyinfo.org, ShouldIScreen.com

## Discussion

To our knowledge, this is the first study to consider health literacy in the context of lung cancer screening. Results of literacy screening among our population of inner city, lower income patients revealed approximately one-third of patients had low health literacy. The relevance of this finding is highlighted by the fact the majority of websites identified in a common search that might be conducted by patients (i.e. what is lung cancer screening?) revealed all educational materials, except one, exceeded the AMA recommendations to be written at or below a 6th grade level. The average Flesch-Kincaid Grade Level of online patient educational materials analyzed was 10.6 while the average American reads at an 8th grade level.

The finding that 38% of patients had low literacy is comparable to results described in similar urban populations who underwent screening for cervical and prostate cancer [35–39]. A pooled analysis of 85 studies found 46% of patients had low or marginal health literacy which was associated with older age, lower education level, and black patients [40]. Low health literacy has been directly linked to worse outcomes across several chronic diseases including, but not limited to, heart disease, diabetes, stroke, advanced kidney disease, asthma, and epilepsy [25, 41–45]. These outcomes may be attributed to limited use of preventative services, higher rates of hospital admissions, increased difficulty managing illnesses, and therefore higher mortality [25, 42, 46, 47].

It is possible the low literacy rate is significantly underestimated in our population. The intake forms were given to patients and they were asked to self-report. It is possible many of the non-responders did not answer the questions due to the stigma of low health literacy and/or due to low health literacy itself. In general, the high rates of low health literacy should raise concerns in a LCS program with a similar population. Recognition of patients with low literacy and optimizing resources to address their educational needs will be critical to the shared decision making process.

In a population similar to our study, lung cancer screening patients preferred information directly from their doctor, and more patients preferred pamphlets/brochures over web-based materials [48]. In the same study, patient knowledge improved with decision aids and 90 min discussion groups. [48]. Another study found improvement in patients’ understanding about lung cancer screening with a shared decision making visit using a six-minute video slide show, a decision aid, and a question/answer session [49]. Though patient knowledge did improve in these studies, not all hospitals have the resources to provide this degree of education during a clinic visit. This can cause anxiety, distress, and confusion leading to a significant portion of medical information being forgotten [50–52]. To mitigate these feelings, patients often perform their own medical research on the internet [19]. Unfortunately, we know online information, regardless of disease topic, is written above the literacy level for most patients [20–23]. It has also been shown older patients, patients with lower household incomes, and those with low health literacy are less likely to use the internet for health research, likely due to their difficulty using a computer [19, 53]. When online material is difficult to comprehend and vulnerable populations have limited access to resources, providing adequate education to those with low health literacy is challenging. Knowledge retention is an additional challenge since this process can take place over several years. Thus, these initial intense interventions may need to be repeated which will be even more resource-consuming.

For patients with low health literacy being referred for LCS to physicians with limited time and resources the best pragmatic option for education is unknown. Our study shows the internet is likely not a viable source. A recent study found similar results indicating most lung cancer screening material is written above the literacy level of the average American [54]. A simpler intervention repeated over time may yield better knowledge transfer and retention in this population. A grade level appropriate pamphlet/decision aid that can be referenced as needed and which simplifies the complexity of lung cancer screening may be a reasonable option. It has been suggested decision aids, though intended for patients, can also improve provider knowledge [13]. When developing educational materials, efforts should be made to keep sentences short, avoid medical jargon, and use simple language associated with pictures. Feedback from target populations is also important when developing educational materials [55].

Our study does have limitations. First, the cohort is from a single urban academic center with a predominantly minority population which limits generalizability. Second, as in any literacy study, there is the potential for non-response bias as patients may feel the questions are too personal or even insulting. Third, the forms used screening questions rather than more involved measurement tools to determine the literacy rate, however, the questions have been validated. Fourth, patients who were eligible for lung cancer screening but did not receive it were not included in the study, therefore the generalizability for all eligible lung cancer screening patients is limited. Fifth, our health literacy screening questions did not contain word labels for response options 2, 3, and 4 on the Likert scale. All the referenced screening questions associated option 3, the middle option, with the word ‘sometimes.’ Therefore, we cannot assume our unlabeled option 3 is equivalent to the label ‘sometimes’ noted in the references. Finally, the focus on English language websites and inclusion of predominately English speaking patients also limits generalizability.

## Conclusion

In our population, low health literacy was common and is likely a barrier to appropriate education for lung cancer screening. The current online educational materials regarding lung cancer screening are written above the recommended reading level set by the AMA, which may further increase patient confusion and anxiety. As more physicians order lung cancer screening for their patients, the need for educational materials directed at those with low health literacy needs to be further explored. Optimizing resources to address these educational needs will be a challenge but is essential to improve the lung cancer screening process.

## Abbreviations

AMA: American Medical Association
FKGL: Flesch-Kincaid Grade Level
LCS: Lung cancer screening
NLST: National lung cancer screening trial
PCP: Primary care physicians
REALM: Rapid estimate of adult literacy in medicine
SES: Socioeconomic status
TOFHLA: Test of functional health literacy in adults
